# Structural health monitoring of jacket-type support structures in offshore wind turbines: A comprehensive dataset for bolt loosening detection through vibrational analysis

**DOI:** 10.1016/j.dib.2024.110222

**Published:** 2024-02-22

**Authors:** Rhandall Valdez-Yepez, Christian Tutivén, Yolanda Vidal

**Affiliations:** a Mecatrónica, Facultad de Ingenierías, Universidad ECOTEC, Samborondón, Samborondón, EC092303, Ecuador; b Mechatronics Engineering, Faculty of Mechanical Engineering and Production Science, Escuela Superior Politécnica del Litoral, ESPOL, Campus Gustavo Galindo Km. 30.5 Vía Perimetral, P.O. Box 09-01-5863, Guayaquil, Ecuador; c Control, Data, and Artificial Intelligence, Department of Mathematics, Escola d'Enginyeria de Barcelona Est, EEBE, Universitat Politècnica de Catalunya, UPC, Campus Diagonal-Besòs (CDB) 08019, Barcelona, Spain; d Institut de Matemàtiques de la UPC, BarcelonaTech, IMTech, Pau Gargallo 14, 08028 Barcelona, Spain

**Keywords:** Structural health monitoring, Experimental, Vibrational analysis, Jacket-type support, Bolt loosening, Structural integrity

## Abstract

This dataset provides a comprehensive collection of vibrational data for the purpose of structural health monitoring, particularly focusing on the detection of bolt loosening in offshore wind turbine jacket foundations. The data set comprises 780 comma-separated values (CSV) files, each corresponding to specific experimental conditions, including various structural states of the wind turbine's support structure. These states are systematically varied considering three main aspects: the amplitude of a white noise (WN) signal, the type of bolt damage, and the level at which damage has occurred.

The data were meticulously collected using eight triaxial accelerometers (PCB R Piezotronic model 356A17), strategically placed at different locations on a scaled-down replica of an offshore jacket-type wind turbine. This setup facilitated the acquisition of detailed vibrational data through a National Instruments’ data acquisition (DAQ) system, comprising six input modules (NI 9234 model) housed in a chassis (cDAQ model). The white noise signal, simulating wind disturbance at the nacelle, was produced by a modal shaker and varied in three amplitudes (0.5, 1, and 2), directly proportional to the induced vibration in the wind turbine.

The dataset uniquely captures the vibrational behaviour under different scenarios of bolt loosening in the turbine's foundation. The conditions include a healthy state (bolts tightened to 12 Nm) and various degrees of loosening (bolts loosened to 9 Nm, 6 Nm, and completely absent), examined at four distinct levels of the turbine's base structure. This granular approach offers a nuanced view of how varying degrees of bolt loosening impact the vibrational characteristics of the structure.

The value of this dataset lies in its potential for wide-ranging applications in the field of structural health monitoring. Researchers and engineers can leverage this data for developing and testing new methodologies for early damage detection and progressive damage assessment in offshore wind turbines. The dataset's comprehensive coverage of damage scenarios makes it a valuable resource for the validation and enhancement of existing damage detection algorithms. Furthermore, the dataset can serve as a benchmark for comparing the efficacy of different vibrational analysis techniques in the context of wind turbine maintenance and safety. Its application is not only limited to wind turbines but can extend to other structures where bolt integrity is critical for operational safety.

This dataset represents a significant contribution to the field of structural health monitoring, providing a detailed and practical resource for enhancing the reliability and safety of offshore wind turbines and similar structures.

Specifications Table

**Table:** 

Subject	Engineering – Mechanical Engineering
Specific subject area	Wind Turbine Structural Health Monitoring
Data format	Raw
Type of data	Time-domain vibration signals (csv files)
Data collection	Data were collected using eight PCB R Piezotronic 356A17 triaxial accelerometers, positioned on a scaled-down offshore wind turbine model. Vibrational data were captured via a National Instruments DAQ system with six NI 9234 input modules in a cDAQ chassis. Experiments simulated varying structural states using three white noise amplitudes (0.5, 1, 2) to mimic different wind speeds, and different bolt conditions (tightened to 12 Nm, loosened to 9 Nm, 6 Nm, and absent) at four levels of the turbine's base.
Data source location	Institution: Universitat Politècnica de Catalunya – BarcelonaTech (UPC) – Escola d'Enginyeria de Barcelona Est (EEBE), Diagonal Besòs Campus City: Barcelona Country: Spain
Data accessibility	Repository name: CORA-RDR: https://www.csuc.cat/ca/serveis/cora-repositori-de-dades-de-recerca Data identification number: 10.34810/data1011 Direct URL to data: https://doi.org/10.34810/data1011 Instructions for accessing these data: Download the Zip file with the dataset by selecting “Access Dataset” in the URL provided.

## 1 Value of the Data

• This dataset provides vibrational data for the analysis of offshore wind turbine foundations under various bolt loosening conditions, comparing them to available data of the healthy case. It portrays how different states of bolt tightness, which cause high vibration levels, affect the structural integrity of wind turbines.
• The data includes measurements obtained at three white noise amplitudes to mimic different wind speeds, allowing for an assessment of the impact of bolt loosening under different environmental conditions. Furthermore, the data could be used for damage detection, localization and isolation, given that it was acquired with triaxial accelerometers at different levels of the jacket structure of a scaled-down wind turbine.
• This dataset is beneficial for structural engineers, researchers in renewable energy, and maintenance teams specializing in offshore wind turbines and developing advanced structural health monitoring systems to ensure safety in renewable energy infrastructures.
• Researchers can use this dataset to develop or validate algorithms for early damage detection and progressive damage assessment in wind turbine foundations. It can also serve as a benchmark for testing the effectiveness of different structural health monitoring techniques and for training machine learning models in predictive maintenance applications.
• This dataset can foster cross-disciplinary research, combining fields like materials science, mechanical engineering, and environmental studies, to explore new solutions for the challenges faced in the renewable energy sector.

## 2 Background

In the evolving field of renewable energy, offshore wind turbines play a pivotal role due to their significant contribution to green energy sources. However, the structural integrity and operational efficiency of these turbines are now crucial concerns, particularly given the high operational and maintenance (O&M) costs, which can account for up to 30 % of the cost of energy (COE) in offshore wind farms [1]. These costs are exacerbated by the turbines' exposure to harsh environmental conditions, which cause several structural damage at different locations in the wind turbine.

To detect those damage, changes in structural dynamics are analyzed via vibration-based statistical time series structural health monitoring (SHM) methods [2]. Particularly, offshore wind turbines in deeper waters (usually between 30 and 90 m)​​ use jacket-type foundations, which are distinct in their structure and require a tailored approach for SHM [3]. However, data acquired during damage states is scarce as a result of frequent maintenance procedures to ensure a constant healthy condition.

Therefore, availability of structural data from damaged states in wind turbines becomes of utmost importance to develop new methodologies aimed at early damage detection and progressive damage assessment in offshore wind turbines. To help address this issue, we created a dataset with a particular focus on the detection and analysis of bolt loosening. This damage type, if neglected, poses substantial maintenance challenges and safety risks in wind turbines [3].

## 3 Data Description

The dataset comprises 780 files in comma-separated values (CSV) format, each file representing an individual experiment that investigates a distinct structural state of the wind turbine's support structure. These structural states are delineated by considering a combination of three variables: the amplitude of a white noise (WN) signal, the type of damage inflicted, and the specific level at which this damage has occurred. The white noise signal, employed to emulate wind disturbances at the turbine's nacelle, is generated through a modal shaker.

Three distinct amplitudes of the white noise signal are utilized in these experiments: 0.5, 1, and 2. The amplitude selected is directly proportional to the level of vibration induced in the wind turbine (WT). Regarding the damage types, the dataset comprises four conditions of a particular bolt in the wind turbine's foundation: a baseline healthy state (bolt tightened to 12 Nm), and three states of loosening (bolt loosened to 9 Nm, loosened to 6 Nm, and completely removed). These loosened and absent conditions are further investigated across four different levels of the turbine's base structure, as depicted in Fig. 4.

Each structural state, defined by a unique combination of white noise amplitude, damage type, and damage level (except in the case of the baseline healthy state), is represented by 20 files in the dataset. Each file corresponds to an experiment conducted over a duration of 60 s. The distribution of experiments across the various structural states is detailed comprehensively in Table 1.

**Table 1:** Number of experiments associated to every experimental case in the dataset.

Structural state	0.5 WN	1 WN	2 WN	Total
Loose bolt at 9 Nm, level 1	20	20	20	60
Loose bolt at 9 Nm, level 2	20	20	20	60
Loose bolt at 9 Nm, level 3	20	20	20	60
Loose bolt at 9 Nm, level 4	20	20	20	60
Loose bolt at 6 Nm, level 1	20	20	20	60
Loose bolt at 6 Nm, level 2	20	20	20	60
Loose bolt at 6 Nm, level 3	20	20	20	60
Loose bolt at 6 Nm, level 4	20	20	20	60
Without bolt, level 1	20	20	20	60
Without bolt, level 2	20	20	20	60
Without bolt, level 3	20	20	20	60
Without bolt, level 4	20	20	20	60
Healthy	20	20	20	60
**Total**	**260**	**260**	**260**	**780**

**Fig. 1:**
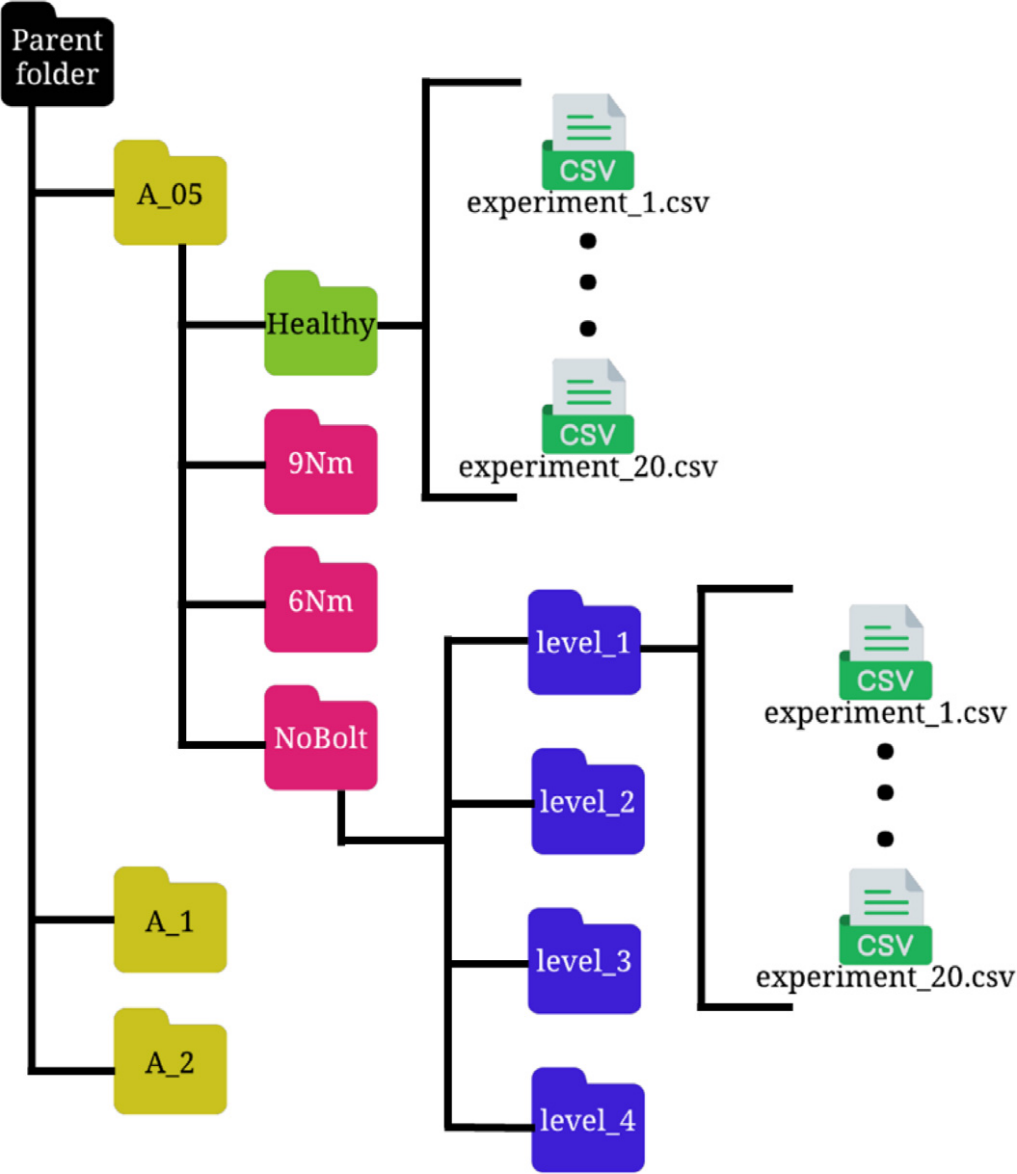
Arrangement of folders and subfolders in the dataset.

The organizational schema of the dataset is meticulously outlined in Fig. 1. The dataset is structured hierarchically within folders, methodically categorized first by the amplitude of the white noise (WN) signal, followed by the type of damage, and concluding with the level at which the damage is present. Within this structure, each specific structural state is represented by 20 comma-separated values (CSV) files, systematically stored in subfolders. These subfolders are nested within parent directories, each layer successively detailing the characteristics of the corresponding structural state.

The root directory of the dataset is divided into three primary folders, with each folder representing one of the WN amplitudes under analysis. The internal structure of these amplitude folders is consistent and can be referenced in Fig. 1, particularly within the folder labeled “A_05”, which denotes the case with a WN amplitude of 0.5.

Within each amplitude-specific folder, there are four subfolders. One of these subfolders, marked in green, pertains to the healthy state of the wind turbine's (WT's) foundation. This folder contains 20 files that encapsulate experiments conducted under specific WN amplitude conditions without any inflicted damage. The remaining three subfolders, indicated in red, are associated with different types of damage. Each red folder is further subdivided into four additional subfolders. Those subfolders correspond to various levels of the WT's base structure. Each subfolder within this category houses 20 files, with each file documenting a 60-second experiment relevant to its designated structural state.

**Fig. 2:**
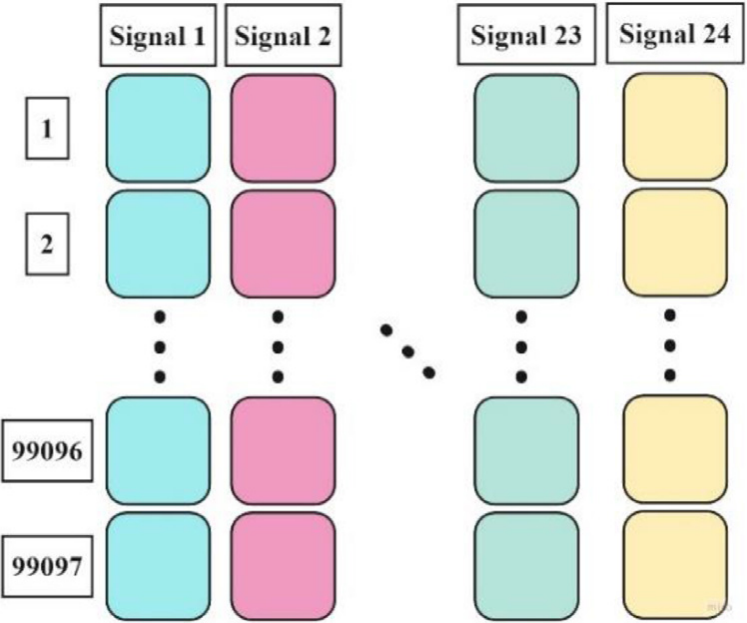
Structure of a csv file.

The composition and format of the data within each comma-separated values (CSV) file are systematically illustrated in Fig. 2. The dataset was generated utilizing eight triaxial sensors, resulting in the acquisition of 24 distinct signals. These signals are each represented by a separate column within the CSV files. The data acquisition system, configured with a sampling frequency of 1.6 kHz, captured a total of 99,097 measurements from each signal over the duration of 60 s for each experiment.

Consequently, the structure of each CSV file is configured as a matrix comprising 24 columns, corresponding to the signals from the triaxial sensors, and 99,097 rows, each row representing a discrete measurement point. This matrix format provides a comprehensive and detailed representation of the vibrational data captured during each experimental condition. The dataset is available in [2].

## 4 Experimental Design, Materials and Methods

### 4.1 Experimental setup

**Fig. 3:**
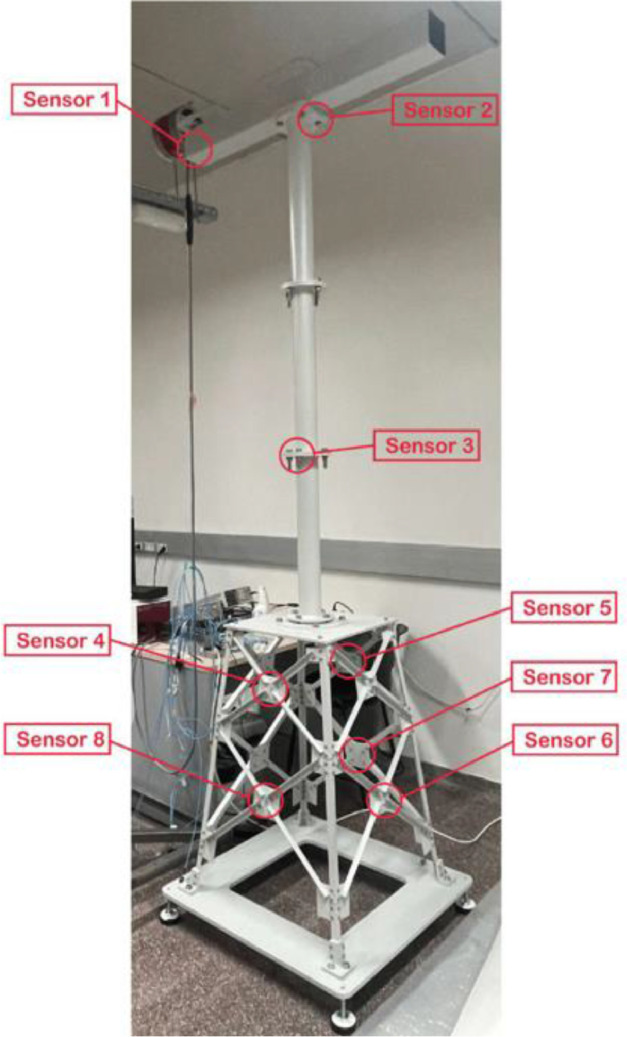
Triaxial sensors’ positions in the wind turbine.

The experiment was conducted using a scaled-down replica of an offshore jacket-type wind turbine. This model was specifically designed to replicate the vibrational characteristics of an actual wind turbine's support structure under various conditions. Fig. 3 illustrates the replica wind turbine, measuring 2.7 m in height, and is comprised of three distinct components:

- The top beam (1 × 0.6 m), to which the modal shaker is affixed, simulating the mass of a nacelle and the effects of wind excitation.
- The tower, consisting of three tubular sections joined by bolts.
- The jacket structure, which is a pyramidal assembly of 32 bars made from S275JR steel in varying lengths, DC01 LFR steel sheets, and other components such as bolts and nuts.

**Fig. 4:**
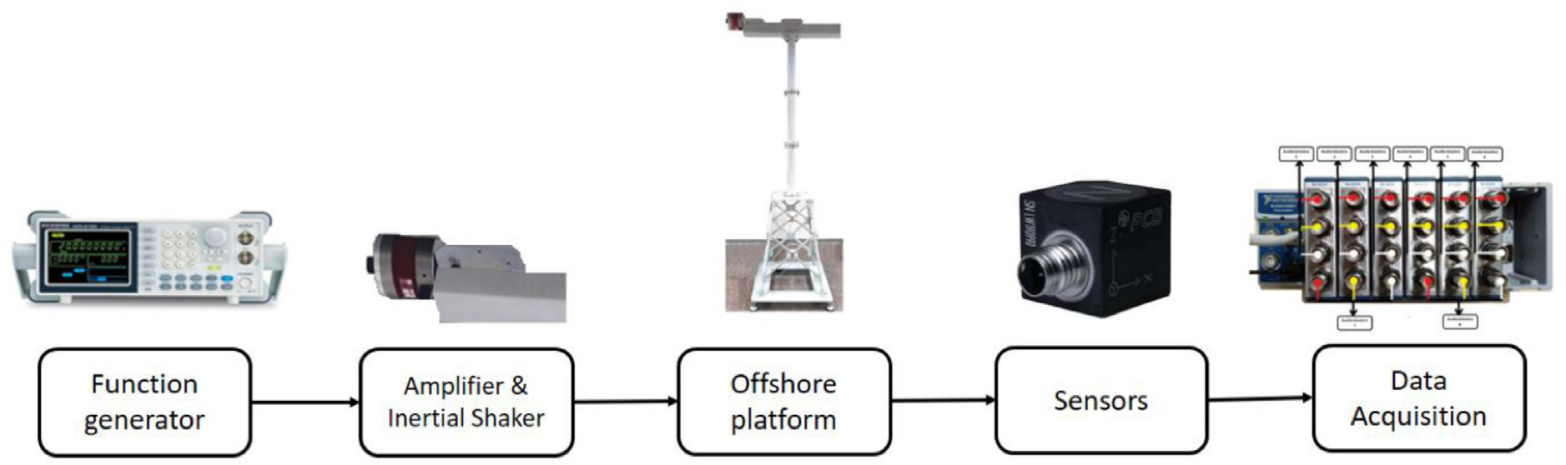
Experimental testbed schematic overview [4].

Fig. 4 presents a detailed schematic of the experimental testbed. The testing procedure commences with the generation of a white noise signal by a function generator. This signal is amplified and relayed to an inertial shaker, which is instrumental in producing vibrations akin to those experienced by a wind turbine under steady wind conditions. The shaker, strategically positioned at the upper segment of the laboratory tower structure, effectively simulates the mass of the nacelle. Vibrational responses of the structure are meticulously captured by eight triaxial accelerometers, all interfaced with a sophisticated data acquisition system. Subsequent subsections provide an in-depth description of the testbed's various components and the instrumentation employed.

### 4.2 Instrumentation

In this study, a function generator is utilized. It is a device capable of delivering a voltage output over a predetermined time frame. The specific model employed was the GW INSTEKAF-2005. For conducting the experiments, a white noise signal was chosen. This signal was manipulated to simulate varying wind speeds by adjusting the amplitude at the function generator. The amplitude of the white noise signal was modified by scaling factors of 0.5, 1, 2, and 3, thereby replicating different wind conditions experienced by wind turbines.

**Fig. 5:**
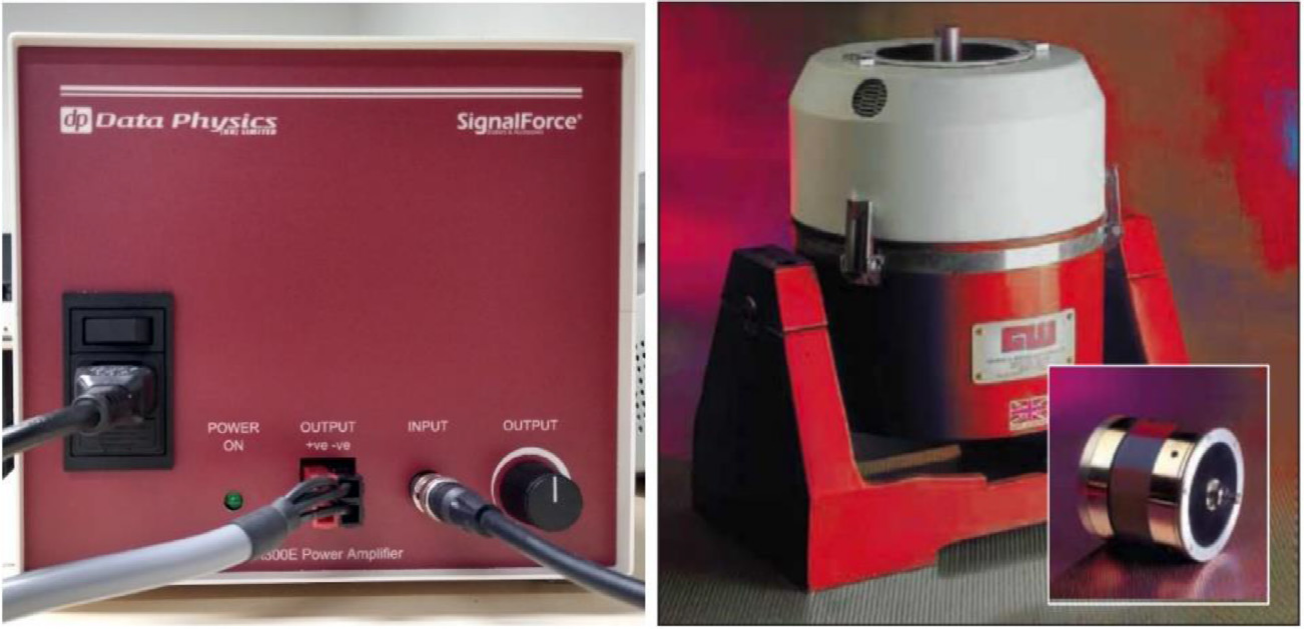
Data physics PA300E amplifier (Left) and IV47 series inertial shaker (Right) [4].

For testing large structures, inertial shakers are the optimal choice. These devices function by attaching their central spigot to the structure being tested, with the body of the shaker acting as the inertial mass. In this study, the inertial shaker model GW-IV47 from Data Physics was employed, alongside its PA300E gain control amplifier, as depicted in Fig. 5. To replicate vibrations similar to those produced when wind impacts wind turbine (WT) blades, the white noise signal from the function generator is amplified. This amplified signal is then fed into the shaker, effectively generating the necessary vibrations to excite the structure under test.

**Fig. 6:**
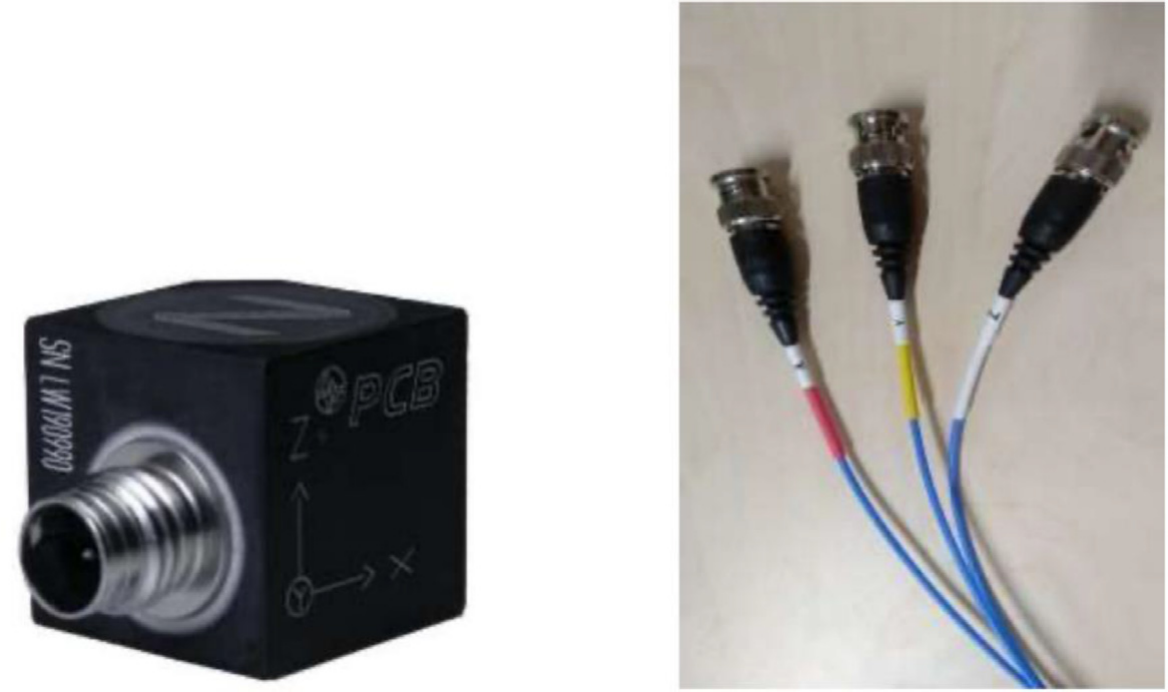
Triaxial accelerometers used in the testbed (PCB Piezotronics, model 356A17) [4].

Data acquisition was performed using eight triaxial accelerometers PCB R Piezotronic model 356A17 (see Fig. 6). Based in [5], these sensors were strategically placed at several key locations on the turbine structure to capture a wide range of vibrational data (see Fig. 3).

The accelerometers were connected to a data acquisition (DAQ) system. This system comprised six input modules (NI 9234 model) housed in a chassis (cDAQ model), all provided by National Instruments. This setup ensured high-fidelity capture of vibrational signals.

### 4.3 Data collection process

The DAQ system was configured to record data at a sampling frequency of 1.6 kHz. This high sampling rate was chosen to ensure that even the most subtle vibrations were accurately captured over the duration of each experiment. Each experiment lasted for 60 s, during which continuous vibrational data were collected from all sensors.

In total, 492 experiments were conducted. These experiments encompassed three distinct white noise (WN) amplitudes, four different structural states, and four levels within the base structure. The experiments were distributed across the WN amplitudes as follows:

- Sixty experiments under healthy conditions, with all bolts in the base tightened to 12 Nm.
- One hundred forty-four experiments, considering each of the four-bar levels with the first type of damage, characterized by a bolt loosened to 9 Nm.
- One hundred forty-four experiments, with the second type of damage, involving a bolt loosened to 6 Nm, at each level.
- An additional one hundred forty-four experiments, considering each of the four levels where the third type of damage involved an absent bolt.

Each experiment yielded 99,097 measurements from every sensor, conducted over a 60-second duration at a sampling frequency of 1.6 kHz. With 8 triaxial accelerometers used, the total number of signals recorded per experiment amounted to 24.

### 4.4 Simulation of different structural states

**Fig. 7:**
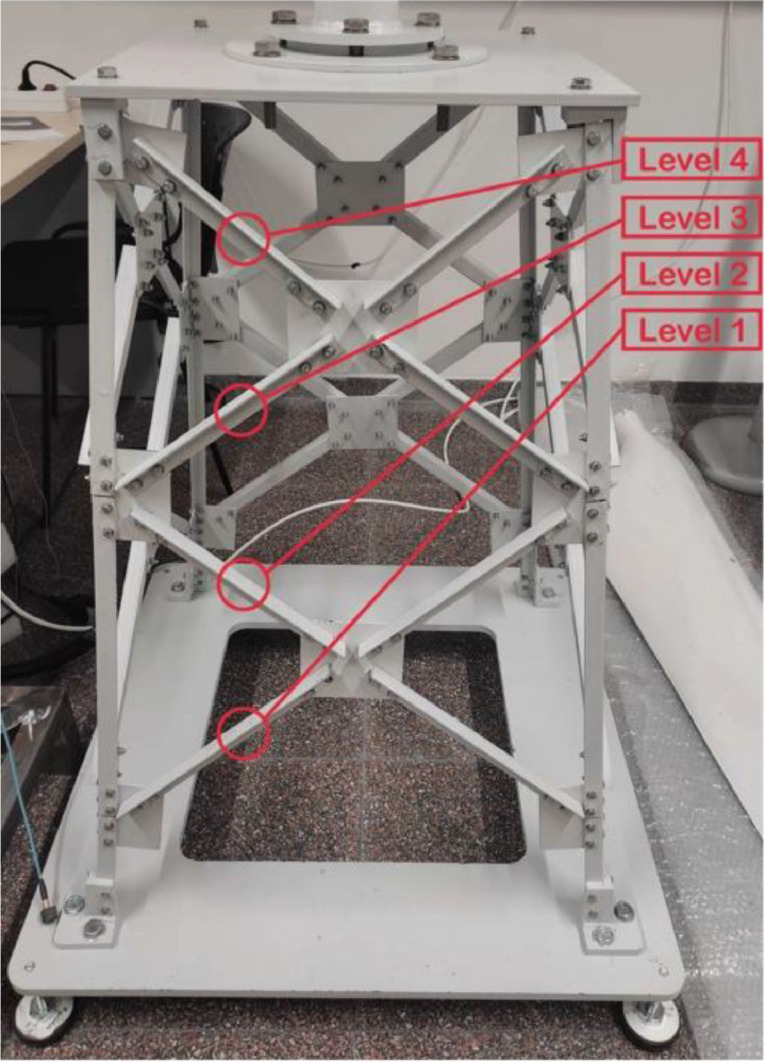
Scaled-down wind turbine foundation's levels.

The experiments were designed to simulate various structural states of the wind turbine's foundation at four different jacket-structure levels (see Fig. 7).

**Fig. 8:**
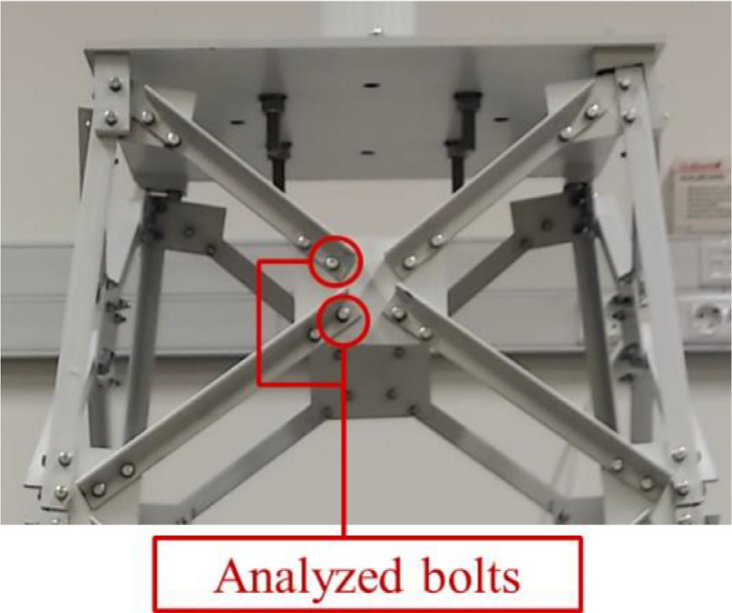
Loosened bolts’ location to replicate damage scenarios.

This was achieved by adjusting the tightness of specific bolts in the structure (see Fig. 8), creating scenarios ranging from fully tightened (healthy state) to different levels of loosening, and ultimately to complete removal (damaged states).

**Healthy State:** In the healthy state, the bolts were secured at a torque of 12 Newton-meters (Nm). This setting was considered the baseline or reference state, representing an ideally intact and fully operational condition of the wind turbine's foundation.

**Loosened States:** Three distinct levels of bolt loosening were simulated to represent various degrees of structural compromise:

- **Slightly Loosened Bolt State:** Bolts were loosened to a torque of 9 Nm. This state was intended to simulate a minor deviation from the optimal structural integrity.
- **Moderately Loosened Bolt State:** Bolts were further loosened to 6 Nm. This intermediate state aimed to represent a more noticeable structural weakening, potentially impacting the turbine's vibrational behavior.
- **Absent Bolt State:** The most extreme condition tested was the complete removal of specific bolts. This state was intended to mimic a scenario where critical fastening elements of the turbine's foundation are missing, posing a severe risk to structural stability.

The structural states were further diversified by applying white noise (WN) signals at three different amplitudes (0.5, 1, and 2) using a modal shaker. These signals were intended to mimic real-world wind disturbances acting on the turbine.

### 4.5 Data format and structure

The data collected from each experiment were stored as comma-separated values (CSV) files. Each file represented a unique combination of WN amplitude and bolt condition.

The files were organized hierarchically in a directory structure based on the amplitude of the WN signal, type of bolt damage, and the level of the turbine's base structure where the damage occurred.

### 4.6 Experimental conditions

All experiments were conducted under controlled laboratory conditions. Ambient environmental factors such as temperature and humidity were monitored and maintained at consistent levels to ensure that they did not influence the data.

### 4.7 Data integrity and verification

To ensure the accuracy and reliability of the data, calibration checks were routinely performed on all sensors and the DAQ system. Additionally, a subset of the data was periodically reviewed and verified for consistency and quality.

Previously described data was collected from a series of sensors arranged at different positions along a scaled-down wind turbine.

## Limitations

The implemented materials, as well as the experimental design, were determined considering acquired data would be employed for proofs of concept regarding designed methodologies for structural analysis of wind turbines with jacket-type foundations.

In the experimental design, wind-induced vibration is the only external disturbance considered, being simulated at a specific location within the structure. However, this approach does not encompass additional environmental factors that can contribute to structural vibrations. Oscillations resulting from wave interactions with the foundation, collisions with external objects, and hydrodynamic pressures beneath the sea's surface, especially due to tidal forces, are not included. Variations in vibrational signals might also be influenced by other external factors, such as ice accumulation on the structure or changes in sea level. Therefore, the data acquired, while representative of wind-induced vibrations, is not fully indicative of the complex spectrum of structural vibrations encountered in real-world conditions. This limitation suggests that the study is at an intermediate Technology Readiness Level (TRL), pointing to the necessity for further experimental research that incorporates a broader range of environmental factors, to advance the TRL and make the findings more applicable to real-world scenarios.

Experimental cases with all the types of damage have been overrepresented in the dataset with respect to the cases where the wind turbine is in a healthy structural state. Hence, the dataset is originally biased.

## Ethics Statement

The authors have read and followed the ethical requirements for publication in Data in Brief and confirm that the current work does not involve human subjects, animal experiments, or data collected from social media platforms.

## CRediT authorship contribution statement

**Rhandall Valdez-Yepez:** Data curation, Investigation, Software, Writing – original draft, Writing – review & editing. **Christian Tutivén:** Investigation, Supervision, Methodology, Writing – review & editing. **Yolanda Vidal:** Resources, Writing – review & editing, Project administration, Funding acquisition.

## Data Availability

Structural health monitoring of jacket-type support structures in offshore wind turbines: a comprehensive dataset for bolt loosening detection through vibrational analysis (Original data) (Dataverse).
